# Hyperbilirubinemia in normal healthy donors

**DOI:** 10.4103/0973-6247.53875

**Published:** 2009-07

**Authors:** Veena Arora, R. K. Kulkarni, Susan Cherian, Raji Pillai, M. Shivali

**Affiliations:** *Blood Bank, Pathology Department, B.A.R.C Hospital, Mumbai – 400 094, India*

**Keywords:** Asymptomatic, donors, Gilbert’s, hyperbilirubinemia, unconjugated

## Abstract

The present study was carried out in B.A.R.C. Hospital Blood Bank over a span of five years, and includes 2734 donors. All the bags were screened for HIV, HBsAg, HCV and VDRL and the plasma in the pilot tubes of the blood bags was observed to detect any abnormality in color. In 27 cases plasma was found to be icteric and liver function tests were carried out on these samples. Two donors showed higher SGPT level, and were excluded. No significant increases in liver enzymes were recorded in the others. Causes of icteric plasma in these apparently healthy donors are discussed. Differential diagnosis includes Gilbert’s disease, hemolytic anemia, drug-induced anemia and other hepatic causes of hyperbilirubinemia, of which Gilbert’s disease is most probable cause with a prevalence of 0.91% in our population. As there are no studies to document the safety of the recipients receiving such abnormal colored plasma as well as to document the hazards in its transfusion, the question arises whether to transfuse such units or not. This study highlights this dilemma. A reassessment of existing policies and regulations is merited.

## Introduction

An elevated level of bilirubin in blood is termed as hyperbilirubinemia. Normal serum bilirubin concentration ranges from 0.1 - 1.2 mg/dl, most of which is unconjugated (0.1 to 1.0 mg/dl).[1] A mild rise of bilirubin between normal and 2 mg/dl is generally not accompanied by visible jaundice. All units of donated blood are being screened for infectious diseases i.e. HIV infection, hepatitis B infection, hepatitis C infection, syphilis and malaria. However, some seronegative donated blood shows high colored plasma with mild increase in unconjugated bilirubin level in asymptomatic donors. No guidelines exist, as to whether such blood bags can be issued to patients or should be discarded. This study has been done to find the prevalence of mild hyperbilirubinemia in seronegative healthy donors in our population and to discuss the possible etiology.

## Materials and Methods

The present study was carried out in BARC Hospital Blood Bank. Donors’ blood collected from 1^st^ January 2001 to 31^st^ December 2005 was analyzed. All bags collected in this period were routinely screened for HIV antibody, HBsAg, HCV antibody, VDRL test and test for malarial parasite. Routine history, physical examination and recording of vital parameters were done for each donor. Donors were deferred as per FDA and SBTC guidelines. This study included only whole blood donation, from donors who were negative for the above tests. Donors were bled aseptically as per FDA and WHO guidelines; time for collection not exceeding 8 minutes, and taking precautions to avoid hemolysis of blood collected. A total of 2793 donors were bled of which 59 (2.11%) seropositive bags were discarded. Hence the study included 2734 asymptomatic donors.

Out of 2734, a few whole blood bags were found to have high colored plasma on standing for 24 hours. Pilot tubes collected from these donors also showed icteric serum [Table 1].

**Table 1:** Whole blood donation over 5 years

Years	Total no. of donor collected	Icteric Bags [%]
2001	483	02 [0.41]
2002	487	04 [0.82]
2003	536	12 [2.24]
2004	685	07 [1.02]
2005	543	02 [0.37]
5 years	2734	27 [0.99]

In our study, 0.99% donors had icteric serum. All these donors were clinically non-icteric, viral marker studies were negative, thus ruling out viral hepatitis and they did not give past history of jaundice. Serology kits used were Biorad (MonoEliza HBsAg ultra, Genscreen HIV plus Ag and Ab, monoEliza anti-HCV plus, HEV and HAV). No significant drug history could be elicited from these donors. Liver function tests were performed on all these samples on Ciba-Corning Expressplus autoanalyzer and findings were noted. Two donors had significant elevation of SGPT, and were excluded from the study. In the remaining, there was no significant derangement of LFT [Table 2].

**Table 2:** Investigations done on the icteric samples

Donor	Total SB	Direct SB	Indirect	SGPT	SGOT	Sr. ALP
	(mg/dl)	(mg/dl)	SB	(U/L)	(U/L)	(U/L)
1/2001	2.01	0.5	1.51	48	39	50
2/2001	1.9	0.4	1.5	59	40	61
3/2002	1.8	0.6	1.2	57	50	54
4/2002	2.3	0.2	2.1	14	15	64
5/2003	2.0	0.2	1.8	48	39	74
6/2003	1.6	0.4	1.2	45	37	63
7/2003	1.7	0.4	1.3	38	35	80
8/2003	1.9	0.4	1.5	42	39	83
9/2003	1.9	0.3	1.6	10	07	50
10/2003	1.8	0.3	1.5	09	12	56
11/2003	1.7	0.4	1.3	21	13	68
12/2003	1.6	0.3	1.3	16	28	33
13/2003	1.9	0.4	1.5	33	29	105
14/2003	3.1	0.3	2.8	09	14	68
15/2003	1.9	0.3	1.6	30	35	45
16/2003	3.1	0.3	2.8	64	26	40
17/2004	2.3	0.3	2.0	34	30	107
18/2004	2.8	1.2	1.6	36	48	56
19/2004	2.8	0.3	2.5	26	33	73
20/2004	2.4	0.3	2.1	20	31	99
21/2004	2.2	0.4	1.8	12	28	100
22/2004	3.1	0.3	2.8	49	54	81
23/2004	2.3	0.3	2.0	19	21	94
24/2005	2.3	0.2	2.1	31	63	87
25/2005	2.8	0.5	2.3	32	63	105

Normal Values: Sr Bilirubin (total): 0.1 - 1.2 mg/dl; Indirect Bilirubin: 0.1 to 1.0 mg/dl; SGOT:8-33 U/L; SGPT:5-40 U/L; Alkaline phosphatase:20-130 U/L (at 37.0 C), Alanine aminotransferase -(ALT or SGPT); Aspartate aminotransferase - (AST or SGOT); and Alkaline phosphatase- (ALP).)

## Results

Out of a total of 2734 donors, 27 donors’ plasma was found to be high colored. Investigations done in these samples include liver function tests i.e. serum total bilirubin, serum unconjugated bilirubin, liver enzymes including SGOT, SGPT, alkaline phosphatase and LDH. Two patients had significant elevation in the liver enzyme levels, but no viral markers were positive, no history of drug or alcohol ingestion was elicited. These two patients were excluded from the study.

Serum alkaline phosphatase levels and LDH levels were within normal limits in all these donors. The increase in serum bilirubin was due to increase in unconjugated bilirubin level. The serum total bilirubin levels in these donors ranged from 1.6 to 3.1 with a mean level of 2.19 mg/dl. Serum unconjugated bilirubin levels ranged from 1.2 to 2.8 mg/dl with a mean level of 1.82 mg/dl Table 2

## Discussion

The observations were analyzed for the prevalence of hyperbilirubinemia. All these donors were clinically non-icteric; viral marker studies were negative. Donors did not give any history of earlier episodes of jaundice. No history of recent drug intake was elicited. Jaundice is one of the criteria for rejection of blood donors. But detection of hyperbilirubinemia in post-donation pilot sample raises a question regarding its etiology. “What could be the possible cause of hyperbilirubinemia in otherwise normal healthy donors?”

Various causes in these cases could be considered.

- Congenital hyperbilirubinemia.
- Drug-induced hemolysis.
- Hemolytic anemia
- Primary liver pathology including the various causes of hepatitis

Out of 27 donors whose units were found icteric, only a few donors were BARC beneficiaries and their previous medical records were studied in detail to get any past history of jaundice or intake of drugs. One donor’s file showed that he was diagnosed as Koch’s 5 years back and had therefore taken anti-tubercular drugs. AKT was however discontinued 4 years back post therapy, thus ruling out a possibility of drug-induced hemolysis. The other reasons for drug-induced hyperbilirubinemia in donors could be ingestion of non-allopathic medicines. Most of the donors feel that ingestion of homeopathic or ayurvedic drugs is not significant and thus deny any history of medication on being asked.

In the rest of the donors, no recent history of drug intake was present. No past history of episodes of jaundice was present. No family history of recurrent episodes of jaundice or any known hemolytic disorders could be elicited. Hence the diagnosis of congenital hyperbilirubinemia or Gilbert’s Syndrome (GS) is very strongly suspected. This chronic disease is characterized by mild persistent unconjugated hyperbilirubinemia. The patient usually does not manifest this disease until after the second decade[2] and is symptomless and unaware of disease until it is detected by routine laboratory testing for other reasons. GS is the most common hereditary cause of increased bilirubin, and is found in up to 5% of the population (though some gastroenterologists maintain that it is closer to 10%).[2] The main symptom is otherwise harmless jaundice which does not require treatment; caused by elevated levels of unconjugated bilirubin in the bloodstream (hyperbilirubinemia).[2] Jaundice is exacerbated following prolonged fasting, surgery, fever or infection and excessive exertion etc. or alcohol ingestion. The source of this hyperbilirubinemia is reduced activity of the enzyme glucuronyl transferase which conjugates bilirubin and some other lipophilic molecules.[3] Conjugation renders the bilirubin water-soluble, after which it is excreted in bile into the duodenum. Liver enzymes and serum LDH are within normal limits in this condition. Hence a significant number of our donors with hyperbilirubinemia might be cases of Gilbert’s Syndrome.

A similar study by Koul *et al*.[4] was conducted in Kashmir, in 1000 randomly selected blood donors. Those with hyperbilirubinemia were further evaluated for a cause of jaundice. Thirty (3%) blood donors had evidence of Gilbert’s Syndrome. All were previously unaware of a hyperbilirubinemic state. Mean serum bilirubin levels were 2.64 mg/dl. The mean serum bilirubin level in our study is 2.19 mg/dl.[4] No other cause of jaundice could be identified upon investigation. They have concluded that Gilbert’s syndrome is seen in 3% of healthy voluntary blood donors in Kashmir. As per our study the prevalence of hyperbilirubinemia in asymptomatic healthy donors in our population is 0.91% (25/2734).

Drug-induced hemolysis may occur in individuals on certain drug therapy. A particular condition called glucose-6-phosphate dehydrogenase deficiency, an X-linked recessive hereditary disease, is characterized by abnormally low levels of glucose-6-phosphate dehydrogenase enzyme (abbreviated G6PD or G6PDH).[5,6] It is a metabolic enzyme involved in the pentose phosphate pathway, especially important in red blood cell metabolism.[7] Individuals with the disease may exhibit non-immune hemolytic anemia in response to a number of causes; develop anemia, jaundice and symptoms of hemolysis especially when there is a positive family history. Our donors did not give a positive family history. Hemoglobin levels were above 12.5m% and LDH levels were within normal limits in all donors. Hence a diagnosis of hemolytic anemia due to G6PD deficiency or drug induced is unlikely.

Causes of hemolytic anemia including thalassemia trait, sickle cell trait, and other causes of immune and non-immune hemolytic anemia have to be considered. Our donors did not give any positive history of any such disease in the family nor did they themselves suffer from recurrent episodes of jaundice and had LDH levels within normal limits. Further workup of these donors is necessary to definitively rule out these conditions.

Hepatitis can be excluded by blood samples negative for antigens specific to the different hepatitis viruses. Cholestasis can be excluded by the absence of elevated alkaline phosphatase level and low levels of conjugated bilirubin. Other causes of a primary hepatic disorder are excluded as SGOT, SGPT and albumin level are within normal ranges. In our donors, liver enzymes were not elevated. More severe types of glucoronyl transferase disorders like Crigler-Najjar syndrome (types I and II) are associated with more severe hyperbilirubinemia and cause brain damage in infancy (type I) and teenage years (type II).

## Conclusion

Existing rules and regulations prohibit issue of blood and components if plasma is abnormal in color. However various authorities have claimed absence of problems in recipients receiving blood with high colored plasma. We have a prevalence of 0.91% for asymptomatic hyperbilirubinemia in healthy donors. We are not willing to expose our recipients to any complication related to blood transfusion. As a result we follow a policy of discarding blood bags with icteric plasma. No pre-donation signs or symptoms can elicit the condition of mild hyperbilirubinemia. Hence strict implementation of deferral guidelines will not solve the problem which includes wastage. Until studies supplemented by adequate data are conducted to confirm the safety of recipients receiving blood with abnormal colored plasma, we will continue to follow this policy. It is up to the regulatory agencies of the blood bank to decide whether screening tests of blood bags should also include serum bilirubin levels. A reassessment of existing policies and regulations is merited.
